# Concept of inverted refractive-index-contrast grating mirror and exemplary fabrication by 3D laser micro-printing

**DOI:** 10.1515/nanoph-2023-0283

**Published:** 2023-08-29

**Authors:** Emilia Pruszyńska-Karbownik, Daniel Jandura, Maciej Dems, Łukasz Zinkiewicz, Artur Broda, Marcin Gębski, Jan Muszalski, Dušan Pudiš, Jan Suffczyński, Tomasz Czyszanowski

**Affiliations:** Institute of Experimental Physics, Faculty of Physics, University of Warsaw, Warsaw, Poland; Department of Physics, Faculty of Electrical Engineering and Information Technology, University of Žilina, Žilina, Slovakia; Institute of Physics, Lodz University of Technology, Łódź, Poland; Institute of Microelectronics and Photonics, Łukasiewicz Research Network, Warsaw, Poland; University Science Park of the University of Žilina, Žilina, Slovakia

**Keywords:** nanophotonics, polymer photonics, subwavelength grating, 3D laser lithography, 3D laser micro-printing

## Abstract

Highly reflective mirrors are indispensable components in a variety of state-of-the-art photonic devices. Typically used, bulky, multi-layered distributed Bragg (DBR) reflectors are limited to lattice-matched semiconductors or nonconductive dielectrics. Here, we introduce an inverted refractive index-contrast grating (ICG) as compact, single-layer alternative to DBR. In the ICG, a subwavelength one-dimensional grating made of a low-refractive-index material is implemented on a high-refractive-index cladding. Our numerical simulations show that the ICG provides nearly total optical power reflectance for the light incident from the side of the cladding whenever the refractive index of the grating exceeds 1.75, irrespective of the refractive index of the cladding. Additionally, the ICG enables polarization discrimination and phase tuning of the reflected and transmitted light, the property not achievable with the DBR. We experimentally demonstrate a proof-of-concept ICG fabricated according to the proposed design, using the technique of sub-µm 3D laser lithography in which thin stripes of IP-Dip photoresist are micro-printed on a Si cladding. This one-step method avoids laborious and often destructive etching-based procedures for grating structuration, making it possible to implement the grating on any arbitrary cladding material.

## 1 Introduction

**Figure 1:**
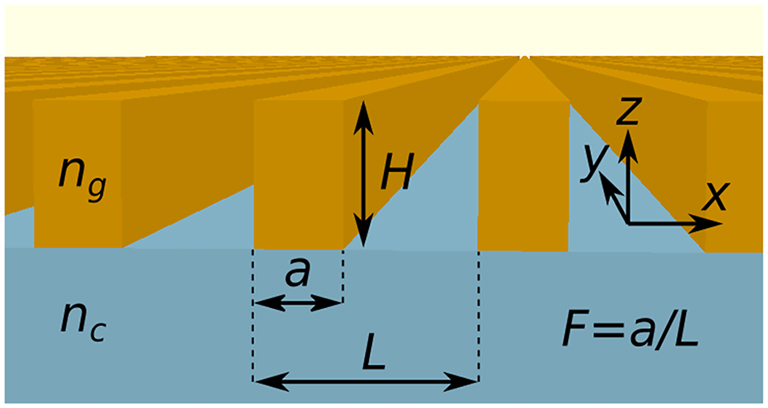
Configuration of the grating structure, composed of stripes with refractive index *n*_*g*_ implemented on a cladding with refractive index *n*_*c*_ and covered by air from the top. In the case of *n*_*g*_ < *n*_*c*_ the grating is the inverted refractive-index-contrast grating (ICG). When *n*_*g*_ > *n*_*c*_ the grating represents a conventional high refractive-index-contrast grating (HCG) and when *n*_*g*_ = *n*_*c*_ the grating is a monolithic high refractive-index-contrast grating (MHCG). The geometrical parameters of the grating and the coordinate system are indicated.

Reflective elements are key components in photonic structures and optoelectronic devices that enhance light–matter coupling effects [1–7]. Conventional high reflectance mirrors employ distributed Bragg reflectors (DBRs) composed of numerous pairs of layers with quarter-wavelength optical thicknesses and contrasting refractive indices. The epitaxial growth of most semiconductor-based DBRs is technically challenging, because of the low refractive index contrast between materials with similar lattice constants. In turn, distributed Bragg reflectors made of dielectric materials [8] suffer from high thermal resistivity, absence of electrical conductivity, and narrow bands of high transmission that make optical pumping difficult. Optical subwavelength structures offer an attractive alternative to multilayer, several-micrometer thick DBRs [9, 10]. A notable example is the high refractive index contrast grating (HCG) [11–13]. An HCG consists of parallel, thin high-refractive-index stripes that are embedded in a low refractive index surrounding. The stripes can be placed on top of a thick layer (Figure 1) made of a low refractive index material, which we call the cladding [11, 14], or suspended in air (i.e., a striped membrane) [15]. A high optical reflection (*R* close to 1) of the HCG results from the destructive interference of the grating modes, which are confined due to the low refractive index of the surrounding [16]. The advantages of HCGs include also a broadband reflection spectrum being up to two times wider spectrally than that of semiconductor DBRs [17], and properties that are impossible to achieve using DBRs, such as strong polarization discrimination and phase tuning of reflected light. On the other hand, the fabrication of HCGs involves a multistep procedure, as it typically relies on electron-beam lithography, photolithography, or nanoimprinting. Using these methods, a stripe-like pattern is defined in a photoresist deposited on the surface of the cladding, followed by metal deposition and lift-off to form a protective mask, before the final step of “wet” or “dry” etching [18].

In our work, we introduce a new design for a highly reflecting subwavelength grating: an inverted refractive index contrast grating (ICG). The design consists of low refractive index grating stripes (*n*_*g*_) deposited by 3D laser micro-printing on a high refractive index cladding layer (*n*_*c*_, Figure 1). Thus, the low and high refractive indices are *inverted* (*n*_*g*_ < *n*_*c*_) with respect to a conventional HCG (*n*_*g*_ > *n*_*c*_).

We start with theoretical analysis indicating the possibility of high reflectance, even though the low refractive index of the grating precludes waveguiding, crucial in the case of HCGs. Next, we verify the theoretical findings by numerical analysis (see Section 6.1). For this purpose, we choose a refractive index of the cladding equal to 3.5 and consider two values for the grating refractive index: 1.5 and 2. The value of 1.5 is very low compared to the refractive index of materials typically implemented in reflecting metastructures. Despite that, we demonstrate a significant level of reflection and point out that the low refractive index grating is suitable for the use as a mirror in resonant cavities designed for sensing, enhancement of the spontaneous emission rate, or producing nonlinear effects. The choice of a grating refractive index of 2 is motivated by the demonstration of reflection into the zeroth diffraction order reaching nearly 100 %, which enables the realization of mirrors for a very broad range of applications in photonics and optoelectronics [19]. We verify our theoretical and numerical analysis by comparison with experimental reflection spectra of an ICG with a very low refractive index grating, which was 3D micro-printed using a photoresist polymer (IP-Dip) on silicon cladding. 3D laser lithography is a versatile alternative to subtractive, multistep, etching-based techniques for producing 3D microstructures [20]. It has been used effectively for the fabrication of various light-harnessing structures, such as 3D photonic crystals, micro-waveguides, and micro-optical elements, including micro-lenses and miniaturized multi-lens objectives [21–34]. However, there are almost no previous reports of using 3D laser lithography on semiconductors to produce subwavelength optical elements. This is mainly due to the common belief that the low refractive indices of polymers used for the printing (from 1.5 to 1.58 [35]), as well as the limited spatial resolution of 3D polymer micro-printing, preclude the application of the 3D micro-printing for the fabrication of reflecting subwavelength gratings. With our work, we show that the 3D laser lithography delivering sub-μm periodic structures is in fact very well suited for the fabrication of subwavelength-type reflecting elements.

## 2 Regimes of reflection in inverted- and high refractive index contrast gratings

**Figure 2:**
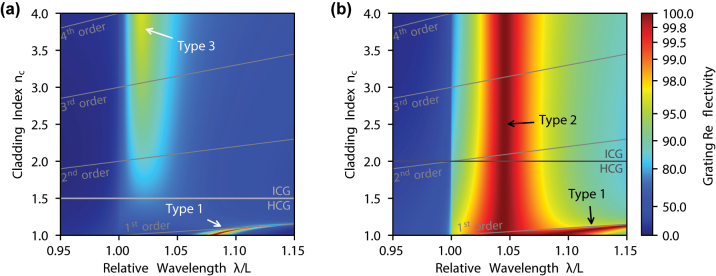
Reflectivity of ICGs with refractive index *n*_*g*_ = 1.5 (a) and 2.0 (b) calculated as a function of the relative wavelength (*λ*/*L*) and the cladding refractive index *n*_*c*_. The other grating parameters are as follows: *H*/*L* = 0.468, *F* = 0.395 and *H*/*L* = 0.373, *F* = 0.414 for (a) and (b), respectively. *H* denotes height, *L* – period, and *F* – fill factor of the grating. The light incidents from the cladding (bottom) side. There are three qualitatively different mechanisms responsible for high-reflectivity peaks marked in the graphs as Type-1 and 2 (see text). Grey lines indicate the cut-offs of the higher-diffraction orders in the cladding.

**Figure 3:**
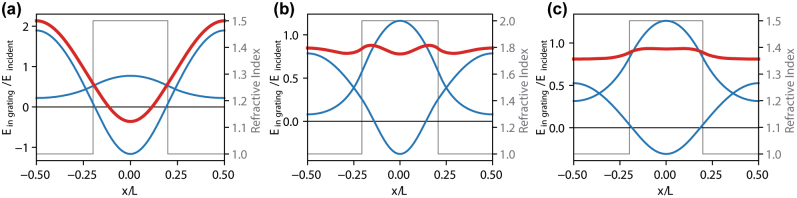
Interference of the grating modes on the input side for high reflectivity peaks of Type 1 (a) and Type 2 (b and c). Plots (a) and (b) are computed for *n*_*g*_ = 1.5 (Figure 2a) and (c) for *n*_*g*_ = 2.0 (Figure 2b). The blue curves illustrate calculated profiles of the individual modes of the grating, while the red ones show their superposition. Electric field intensity is normalized relative to the one of the incident wave. For the reflection maxima of Type 2, this superposition is nearly flat, indicating a near-perfect elimination of higher diffraction orders, which is not the case for the reflection of Type 1. Gray lines indicate refractive index profile in the grating.

In general, the high reflectivity of HCGs is a result of the two-mode interference phenomenon [36, 37]. In the subwavelength regime, such gratings support two modes propagating vertically (in the direction perpendicular to the grating plane) that can couple to each other only at the top and the bottom surface of the grating. Total 100 % reflection occurs when there is destructive interference of these modes on the output (top) side of the grating. On the input (bottom) side, their superposition can be arbitrary. In most conventional HCGs, high reflectivity can be obtained only when the refractive index of the cladding (*n*_*c*_) is low enough that only a single diffraction order exists in the reflection [36, 37]. We name the maxima in the reflection spectrum, which are induced by this mechanism Type 1. Examples of these maxima are shown in plots in Figure 2 presenting reflectivity maps of two gratings with different *n*_*g*_, calculated as a function of the relative wavelength (*λ*/*L*) and the cladding refractive index *n*_*c*_ (see Section 6.1 for details of the method). Their spectral positions depend on *n*_*c*_ and they disappear with the appearance of higher diffraction orders in the cladding. This happens because the two grating modes interfere on the input side, such that the resulting wave couples to higher diffraction orders of the reflected wave, while the low refractive index cladding prohibits their propagation. Figure 3a illustrates interference on the grating input side of a two-mode solution based on the analytical model proposed in Ref. [36], for the Type-1 reflection maximum marked in Figure 2a at *n*_*c*_ = 1 and *λ*/*L* = 1.079. The sine-like shape of the superposed field profile indicates strong coupling to the first and higher orders of the reflected wave.

High reflection mechanisms of a nature different than the Type 1 are also possible, such as the maximum shown in Figure 2b for the wavelength *λ*/*L* = 1.046. Based on the same analytical formalism, a single reflection channel related to the zeroth diffraction order can be identified below the first-order diffraction cut-off (Figure 2b) as the only reflection channel existing in this configuration. The presence of such 100 % reflectivity spectral region very weakly depends on the cladding refractive index. With increasing *n*_*c*_, higher diffraction orders of the reflected wave become possible in the cladding, but the fact that the original zero-order reflection remains almost unaffected by the change of *n*_*c*_ together with the conservation of energy implies that no light is scattered into the higher-order cladding modes. An important property of this reflection mechanism, which we call Type 2, is the nearly flat superposition of the grating modes in comparison to reflection Type 1, as Figure 3b illustrates. In this mechanism, the zero-order component of the grating mode Fourier expansion dominates significantly over other components, enabling nearly 100 % reflectivity into the zeroth diffraction order. The mechanism standing behind this phenomenon is explained in more detail in the Supplementary Materials (Section S1).

Although, for the Type-2 reflectivity, the high-order components of the superposed grating mode at the reflection side are small (Figure 3b and c), they are not zero. The lower the grating refractive index is, the higher is their role in the total reflectivity spectrum of the whole grating. In consequence, for low *n*_*g*_, the interference of the grating modes is not fully destructive the value of the total reflectivity starts to depend on *n*_*c*_ and drops significantly below 100 %. This is particularly visible for *n*_*g*_ below approximately 1.75, as will be shown later in this paper. However, the wavelength of the reflectivity peak remains unchanged (see Figure 2a around *λ*/*L* = 1.022), which clearly distinguishes from Type-1 reflectivity.

The theoretical analysis presented in this section demonstrates the physical mechanism responsible for the emergence of very high reflectivity in the case of the subwavelength grating of refractive index lower than that of the cladding. In the following section we characterize the impact of ICG geometry on optical properties using numerical approach.

## 3 Numerical verification of inverted refractive index contrast gratings properties

In this section we numerically calculate the power reflectance of a grating with the refractive index *n*_*g*_ deposited on the surface of the semi-infinitely thick monolithic cladding with the refractive index *n*_*c*_ larger than *n*_*g*_ (*n*_*g*_ < *n*_*c*_). A semi-infinite air superstrate is assumed above the grating (see Figure 1). We consider reflection in the zeroth-diffraction order only and a single period of the grating with periodic boundary conditions, which elongates the grating to infinity in the lateral direction. The normal incidence of the light from the cladding side is assumed. As a reference, we also consider the case of an HCG where *n*_*g*_ > *n*_*c*_, as well as the border case of a monolithic HCG (MHCG), where *n*_*g*_ = *n*_*c*_ [37, 38].

**Figure 4:**
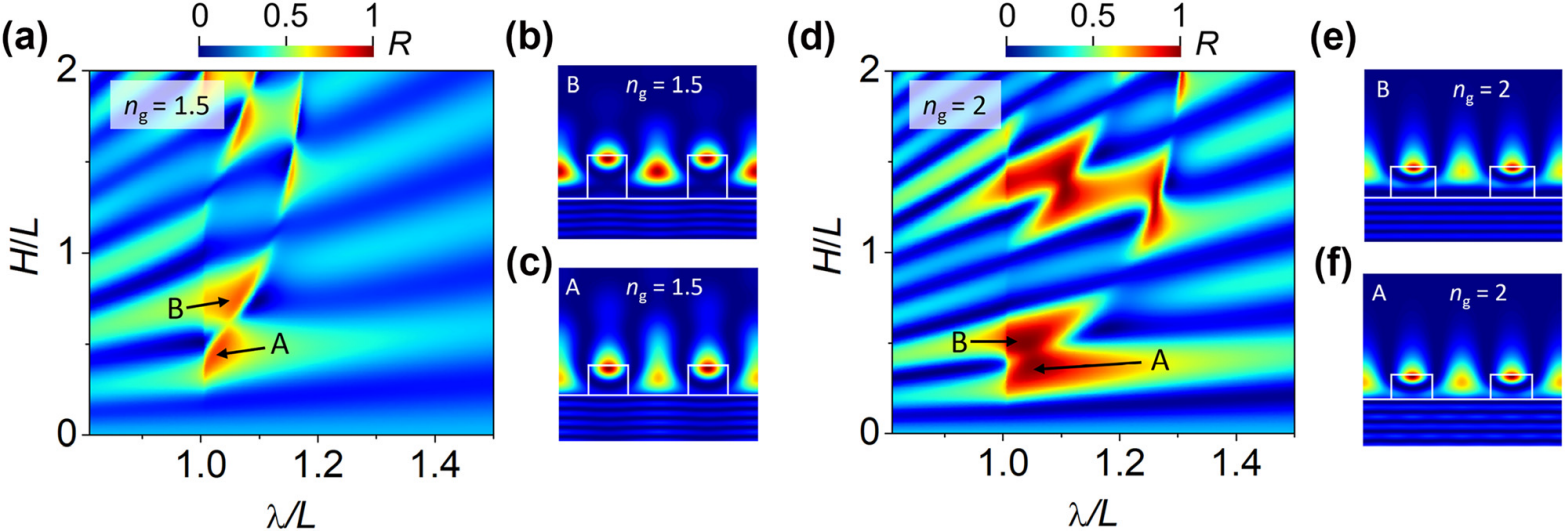
Reflectance map for the inverted contrast grating (ICG) in the domain of wavelength *λ* and grating height *H*, both relative to the grating period *L*, calculated using the PWRTM technique. The refractive index of the cladding *n*_*c*_ is 3.5. The refractive index of the grating and fill factor are assumed as *n*_*g*_ = 1.5, *F* = 0.395 in (a) and *n*_*g*_ = 2.0, *F* = 0.414 in (d). In (b), (c), (e), (f) the distributions of optical field intensity corresponding to the *A* and *B* reflection maxima for *n*_*g*_ = 1.5 (b), (c) and *n*_*g*_ = 2.0 (e), (f) are shown within an ICG illuminated by a plane wave at normal incidence from the cladding side. The parameters of *A* and *B* maxima are collected in Table 1.

Figure 4a and d show maps of power reflectance for two exemplary ICGs. We consider grating material with a refractive index *n*_*g*_ = 1.5 (see Figure 4a) or *n*_*g*_ = 2.0 (see Figure 4d) and the same cladding layer (*n*_*c*_ = 3.5) in both cases. Calculations are conducted in the domain of the height of the stripes (*H*) and the light wavelength (*λ*). Fill factor *F* of the grating is defined as *F* = *a*/*L*, where *a* is a stripe width and *L* is a period of the grating.

The ICG modes are leaky (see Sections 2), whereas HCG grating modes are not. However, the reflection pattern shown in the maps resembles to some extent the “checkboard” pattern observed also in the case of HCG [37]. As shown in Supplementary Materials in Section S2 this region is limited by cutoffs of TE_2*n*_ (from the short wavelength side) and the long-wavelength limit, according to waveguide theory [39]. Above the long-wavelength limit, only TE_0*n*_ modes exist and the grating behaves as a quasi-uniform (unstructured) layer. Therefore, the reflection resembles a Fabry–Perot interference pattern, produced by a uniform layer without any regions of high power reflectance.

Several regions of high power reflectance are visible in both reflection maps (Figure 4a and d), confirming the predictions of the theoretical model presented in Sections 2. In what follows, we focus on the two power reflectance maxima (PRM) that we name *A* and *B* (Figure 4 and Table 1), representing Type 2 mechanism. For these two maxima a width of the reflection stopband (WRS), that we define as a spectral range *λ*/*L* for which the reflection is above 60 %, is the largest.

**Table 1:** Geometrical parameters of the inverted refractive-index-contrast grating (ICG) for configurations corresponding to *A* and *B* maxima for *n*_*g*_ = 1.5 and *n*_*g*_ = 2.0: *L* – period of the grating, *F* – fill factor, *H* – height of the stripe, *R* – optical power reflectance into the 0th diffraction order for *n*_*c*_ = 3.5.

Maximum	*A*	*B*	*A*	*B*
*n*_*g*_	1.5	1.5	2.0	2.0
*λ*/*L*	1.022	1.057	1.046	1.032
*F*	0.395	0.397	0.414	0.447
*H*/*L*	0.468	0.755	0.373	0.502
*R*	0.840	0.810	0.996	1 − 1.8 ⋅ 10^−4^

In the case of the ICG with *n*_*g*_ = 1.5, *A* and *B* PRMs reach more than 80 % (Figure 4a). Both PRMs are located near the mode TE_20_ (see Supplementary Figure S2), which influences the optical field distributions in the grating, contributing to a single optical field maximum along the *z* axis in the region of the grating as illustrated in Figure 4b and c. Increasing the grating refractive index to *n*_*g*_ = 2.0 increases the grating reflectance to nearly 100 % for *A* and *B* PRMs and broadens their WRS, as illustrated in Figure 4d. Light distributions corresponding to *A* and *B* PRMs in the case of *n*_*g*_ = 2.0 are illustrated in Figure 4e and f displaying similar light distribution as in the case of *n*_*g*_ = 1.5. The geometrical parameters of ICG configurations corresponding to *A* and *B* PRMs for *n*_*g*_ = 1.5 and *n*_*g*_ = 2.0 are collected in Table 1. The reflection spectra corresponding to the four maxima are presented in Supplementary Figure S3.

Closer inspection of the light distributions for the PRMs for *n*_*g*_ = 1.5 and *n*_*g*_ = 2.0 reveals that the dominant maximum intensity of the light is located close to the top surface of the stripe. Moreover, the optical field extends into the air above the stripe, independently of its refractive index [40, 41]. There is also a significant build-up of the light density in the grating (see Section S2 in the Supplementary Materials) that may be possibly utilized to enhance light–matter interaction in the region of the grating, as demonstrated previously in Refs. [41, 42]. Light distribution for the *B* maximum for *n*_*g*_ = 1.5 shows additional significant local maxima in the air slit between the stripes (Figure 4b), which could facilitate interaction of the reflected light with the surroundings, enabling possible sensing applications [13, 43]. Further properties of ICGs are discussed in more detail in Section S4 of the Supplementary Materials, while here we will indicate the most important remarks. The first one concerns possibility of high transmission of the light incident from the air side. This property together with the very high reflectance of the zeroth diffraction order when light is incident from the cladding side, is expected to be useful when the ICG constitutes one or both mirrors of a Fabry–Perot cavity subjected to external excitation. Another property of the ICG is possibility of phase tuning of reflected light at the level of d*ϕ*/d*λ* ≈ 10π rad, which provides a facile method of tuning the resonant wavelength of a cavity with an ICG mirror, by modifying the geometrical parameters of the ICG while keeping the cavity thickness constant [44].

**Figure 5:**
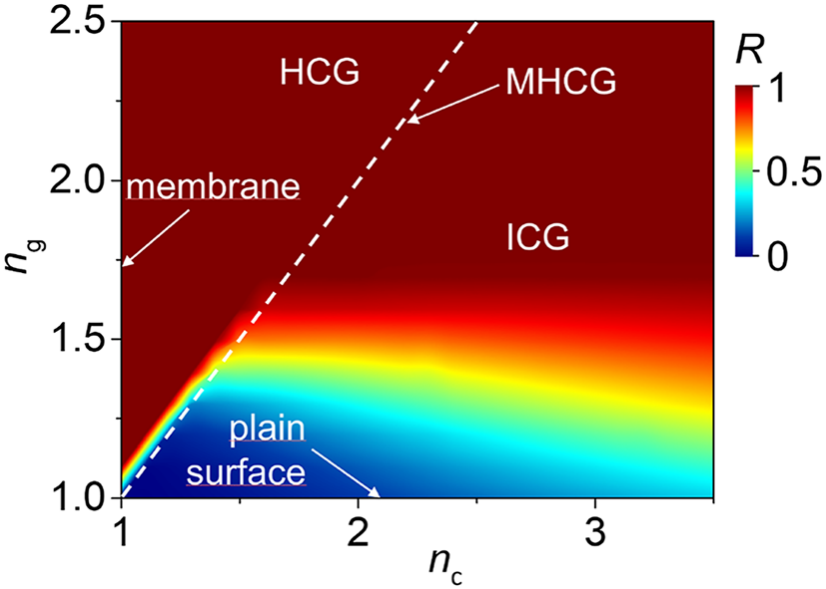
Map of maximal power reflectance for subwavelength gratings calculated in the (*n*_*c*_, *n*_*g*_) space. Each point on the map represents maximal reflection of *B* and *A* PRMs. The white dashed line represents the MHCG configuration (*n*_*c*_ = *n*_*g*_). The region positioned on the left of the dashed line represents an HCG (*n*_*c*_ < *n*_*g*_). The region on the right of the dashed line represents an ICG (*n*_*c*_ > *n*_*g*_). The vertical line at *n*_*c*_ = 1 represents a membrane suspended in air. The horizontal line at *n*_*g*_ = 1 corresponds to a plain surface between the cladding and the air.

A more general picture of the optical performance of the ICG and all possible subwavelength grating configurations is provided in Figure 5, showing the calculated maximal power reflectance into zeroth diffraction order of the gratings in the domain of *n*_*g*_ and *n*_*c*_ for light incident from the cladding side. Magnitude of each point on the map is the largest value for either the *B* or *A* reflection maximum (see Section S6 in the Supplementary Materials). The geometrical parameters of the gratings are modified throughout the map, since modifying the refractive index of grating imposes different conditions for the optimal geometrical parameters ensuring the maximal reflectance. Power reflectance of 100 % into the zeroth diffraction order is achieved by all HCG configurations that fulfil the condition *n*_*g*_ > *n*_*c*_, including the membrane configuration in which the grating is suspended in air (*n*_*c*_ = 1). The MHCG configuration (*n*_*g*_ = *n*_*c*_) enables total reflectance when the refractive index of the grating and the cladding is larger than approximately 1.75, in agreement with Ref. [45]. A previously unexplored feature is an apparent ability of the ICG to achieve nearly 100 % reflection when *n*_*g*_ < *n*_*c*_, which is related to Type 2 reflection as discussed in Section 2. The only requirement is that *n*_*g*_ is larger than 1.75. For *n*_*g*_ > 1.75, the total reflection is found within numerical precision as long as the difference *n*_*c*_ − *n*_*g*_ is less than 0.5, while for *n*_*c*_ − *n*_*g*_ > 0.5 the maximal power reflectance into the zeroth diffraction order is not smaller than 1 − 10^−3^. With a decrease in the refractive index of the grating (*n*_*g*_ < 1.75), the power reflectance and WRS also decrease revealing features of Type 2 reflection (see Section 2). However, as shown in Figure 5, an ICG with *n*_*g*_ < 1.75 still provides power reflectance considerably exceeding the reflectance of the plain surface between the cladding and air. The influence of the refractive index of the grating *n*_*g*_ on the reflection spectrum of ICG is analysed in more detail in Section S6 in the Supplementary Materials.

As discussed above, an HCG in which *n*_*g*_ > *n*_*c*_ ensures total reflection into the zeroth diffraction order and a wide WRS. However, this configuration requires the implementation of a high refractive index material, such as a semiconductor, on a lower refractive index thick layer, for example a dielectric. This impedes the use of such mirrors in resonant optoelectronic devices, including vertical-cavity surface-emitting lasers, due to practical problems with current injection, heat dissipation, and mechanical stability compared with all-semiconductor configurations. Combining two semiconductor layers of different refractive indices to achieve an HCG is also demanding, due to the typically significant difference in the lattice constants of semiconductor materials with sufficiently high refractive index contrast. Eliminating these problems is possible, in principle, by using an MHCG; however, the fabrication of MHCGs remains a challenge. The concept of an ICG in which a lower refractive index grating is deposited on cladding with a higher refractive index can substantially simplify grating implementation, due to the considerable freedom of forming thin dielectric subwavelength structures on top of semiconductor devices. In particular, the dielectric-semiconductor boundary can be a natural etch-stop that enables better control over the etched structure parameters. An ICG composed of semiconductor cladding and a dielectric grating fabricated using dielectrics with refractive indices higher than 1.75, such as TiO_2_ (refractive index in the range of 2.05–2.48 [46]), TaO_2_ (2.08–2.3 [47]), or Si_3_N_4_ (1.98–2.05 [48]), would allow for nearly 100 % reflection into the zeroth diffraction order of the normal incident light from the semiconductor side. If materials of even lower refractive index, such as SiO_2_ (1.4–1.5 [49]) or IP-Dip photoresist (1.5–1.58 [35]) are deposited on an arbitrary semiconductor, reflection into the zeroth diffraction order is expected to reach 85 % and nearly 98 % into all diffraction orders, as will be demonstrated in the next section.

## 4 Experimental demonstration of 3D micro-printed ICG

**Figure 6:**
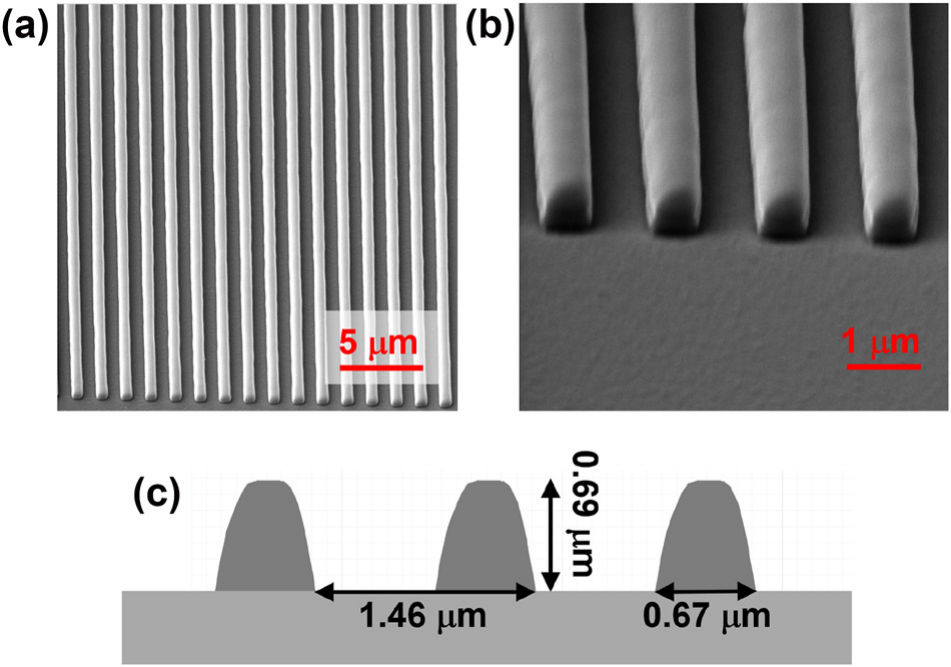
IP-Dip subwavelength grating deposited using 3D laser lithography on a Si cladding: (a, b) scanning electron microscope (SEM) image in two consecutive enlargements; (c) profile of the ICG stripes scanned from SEM images and implemented in the software with the grating dimensions indicated.

To experimentally verify the theoretical model and numerical simulations presented in Sections 2 and 3, we fabricated an ICG employing the 3D laser lithography technique. The low refractive index of IP-Dip photoresist (*n*_*g*_ = 1.53 [35]) prevents 100 % power reflectance into the zeroth diffraction order. However, expected reflectance above 80 % is fully enough in a variety of optical and optoelectronic applications, including resonator cavity enhanced light emitting diodes and resonant cavity enhanced photodetectors. Uniquely, the 3D micro-printing enables flexibility in the fabrication of the ICG, making possible wavelength, phase, and wavefront tuning by tailoring the parameters of the ICG stripes. Figure 6 and Figure S6 in the Supplementary Materials illustrate an ICG fabricated by the 3D laser micro-printing using IP-Dip directly on a thick Si wafer (refractive index of *n*_*c*_ = 3.5 at 1500 nm).

The ICG is designed for peak TE-polarized reflection at *λ* = 1500 nm. The double side polished Si wafer is covered with an antireflective Si_3_N_4_ coating, consisting of a single quarter-wavelength thick layer on the surface opposite to the surface on which the ICG was implemented. The ICG is designed with the following parameters: *λ*/*L* = 1.022, *F* = 0.4, *H*/*L* = 0.47, and *L* = 1460 nm, corresponding to *A* PRM in Figure 4a. The parameters are predicted to provide maximal reflectance assuming a rectangular cross-section of ICG stripes. The process of grating fabrication is detailed in the Fabrication Methods section.

The actual geometrical dimensions of the processed ICGs are determined by scanning electron microscopy (SEM), and *H* is additionally inspected using a confocal optical microscope. For the presented sample, *L* = 1460 nm (determined with 50 nm precision; see Section S7 in the Supplementary Materials), *F* = 0.45 and *H*/*L* = 0.46. To validate the numerical analysis, the actual cross sections of the ICG stripes are extracted from the SEM images (see Figure 6c). The obtained profiles are implemented in the numerical model. In what follows, all numerical results relate to the cross-section shape of the real-world ICG. Reflection maps calculated for the ICG (see Figure S7 in the Supplementary Materials) show great similarity to the reflection maps of an ICG consisting of stripes with a rectangular cross section (see Figure 4a). The deviation in the cross-section in our experiment from the rectangular shape does not affect maximal reflection, but in general it may require modification of the grating parameters to achieve maximal power reflectance [50]. The power reflectance of the ICG with the real-world cross section is discussed in more detail in Section S7 of the Supplementary Materials.

The transmission through the ICG sample can be expressed as follows:

(1a) $$T_{\text{ICG}}=T_{\text{ar}}e^{-\alpha d}\left(1-R_{\text{ICG}}\right)$$

(1b) $$T_{\text{ref}}=T_{\text{ar}}e^{-\alpha d}\left(1-R_{\text{plain}}\right)$$

where *T*_ICG_ is transmission measured for normal incident light through the ICG, *T*_ref_ is the reference transmission through the neighboring unprocessed region of the sample, *T*_ar_ is transmission through the antireflecting coating (which also accounts for any scattering occurring in the wafer and on its surface), *α* is the absorption coefficient of the silicon wafer, *d* is the thickness of the wafer, and *R*_plain_ is the reflection from the plain interface between Si and air (which is 0.304 at the wavelength of 1500 nm based on Fresnel equations for reflection). With the set of Equations (1) the reflectivity of the ICG (*R*_ICG_) can be extracted directly from the transmission measurements:

(2) $$R_{\text{ICG}}=1-\frac{T_{\text{ICG}}}{T_{\text{ref}}}\left(1-R_{\text{plain}}\right)$$

**Figure 7:**
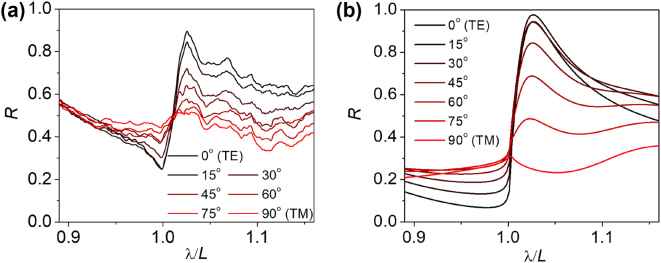
Measured (a) and calculated (b) reflection spectra into all diffraction orders of inverted refractive-index-contrast gratings (ICGs) 3D micro-printed using IP-Dip on a silicon cladding. The spectra are calculated for the geometrical parameters of the stripes determined experimentally, with the grating defined by the following parameters: *L* = 1460 nm, *F* = 0.45, *H*/*L* = 0.46, for polarization modified gradually with a 15-degree step from TE to TM. The TE and TM polarization corresponds to the angle of 0 and 90°, respectively.

Figure 7 presents the measured (Figure 7a) and calculated (Figure 7b) reflection spectra for consecutive angles of the linear polarization from 0 to 90° with a 15° step. The extreme angles of rotation represent TE and TM polarisations. In the measurements, only the zeroth diffraction order is transmitted through the grating, due to the dimensions of the subwavelength stripes. Therefore, *R*_ICG_ accounts for all diffraction orders of light reflected by the grating. The experimental reflection spectra for TE polarisation reveal a local maximum at *λ*/*L* = 1.027 (*λ* ≈ 1500 nm) that agrees very well with the numerical results. The measured maximal reflection is close to 90 %, in line with the numerical simulations, revealing maximal reflection into all diffraction orders at the level of 97 %. The TE reflection abruptly reduces towards shorter wavelengths, which is consistent with the calculated reflection map in Figure 4a, indicating high transmission in this spectral range. Rotation of the polarizer from the position corresponding to TE polarization to the position corresponding to TM polarization reduces the reflectivity to nearly 30 %, which is the level of a reflectance from the interface between silicon and air. At the wavelength corresponding to the maximal reflectance, TE polarisation reflection is twofold larger than TM polarisation reflection. The calculations show that TE polarization reflection can be fivefold larger than TM polarization reflection. The inconsistencies in the measurements and simulations are typically related to the roughness of the side walls of the ICG stripes, which introduce disturbances in the grating periodicity. This issue was extensively discussed in our previous work [50]. The experimental power reflectance can be enhanced by perfecting the process of the ICG fabrication. Overall, our experimental results show very good agreement with calculations and confirm the feasibility of high power reflectance using the ICG design.

## 5 Conclusions

We have presented a new high reflecting mirror design for an inverted refractive index contrast grating, along with a theoretical, numerical, and experimental investigation of its optical performance. By theoretical analysis we demonstrated the possibility of high reflectance independent of the refractive index of the cladding on which the grating is deposited, particularly when the refractive index of the grating is lower than the refractive index of the cladding.

By numerical analysis, we have shown that the ICG provides almost 100 % optical power reflectance for light incident at normal from the side of the cladding layer toward the air. The only requirement is that the grating be formed from a material with a refractive index higher than 1.75. The refractive index of the layer below the grating can be arbitrary. When the refractive index of the grating is less than 1.75, the grating still strongly enhances the power reflectance compared to reflection occurring at the plane interface between the cladding layer and the air. The ICG enables polarization control of reflected light, with a fivefold larger reflection of transverse electric (TE) polarization compared to transverse magnetic (TM) polarization, and facilitates phase tuning of reflected light.

To experimentally verify our numerical analysis, we have fabricated the ICG grating by 3D printing with sub-µm 3D resolution of IP-Dip photoresist on a thick silicon wafer and we have characterized its optical performance. Qualitative and quantitative comparison of measured and calculated power reflectance spectra from the grating revealed very good agreement, indicating nearly 90 % reflection in all diffraction orders and an efficient polarization control.

At a more general level, the proposed design and its implementation using an additive-type technique open up new possibilities for the fabrication of subwavelength structures, which are in increasing demand in the fields of photonics, optics, optoelectronics, and sensing. The fabrication of highly reflective mirrors in the form of 3D micro-printed gratings does not require high-vacuum techniques such as vapor deposition or epitaxy, and has the additional advantage of scalability. Thanks to the relaxation of the requirements for the refractive index of the cladding layer hosting the grating, the range of materials that can be applied is extended, making the use of perovskite or organic grating layers possible.

## 6 Methods

### 6.1 Numerical methods

To determine the optical reflectance of the gratings, we use the plane-wave reflection transformation method [51], which is a fully vectorial optical model. Because of the periodicity of the gratings, the electrical field of the electromagnetic wave can be expressed in the form of Bloch waves: $\Psi \left(x\right)=e^{ik_{x}x}f\left(x\right)$ , where *f*(*x*) is a periodic function with the same period as the grating *L*, and *k*_*x*_ is the lateral component of the wavevector of the light, ranging from −*π*/*L* to *π*/*L*. In the analysis, we use 60 plane waves that enable numerical relative error below 10^−8^. The model has been shown to have high reliability by comparison with experimental results [52, 53]. In the analysis we consider transverse electric (TE) polarization, where the electric field is parallel to the grating stripes. Transverse magnetic (TM) polarization perpendicular to the grating is not considered here, as the ICG shows significantly lower power reflectance of this polarization.

### 6.2 Fabrication methods

To fabricate the ICG grating, we used the Photonic Professional GT laser lithography system from Nanoscribe GmbH with a 63× immersion objective and IP-Dip polymer material. The system uses Er-doped femtosecond frequency-doubled fiber laser emitting pulses at 780 nm wavelength with an approximately 100 MHz repetition rate and 150 fs pulse width. The femtosecond laser is focused into the volume of the IP-Dip photoresist, where the two-photon polymerization process occurs in the volume of the focal spot (voxel). In the fabrication process, the IP-Dip polymer was deposited on top of the silicon substrate and polymerization by laser writing was realized layer by layer in a single-step process. The grating structure on top of the silicon cladding was fabricated using a programmed script in a two-layer arrangement of horizontal stripes, with laser power of 26 mW and a scanning speed of 10,000 μm/s. For the development of a polymerized structure, PGMEA (propylene glycol monomethyl ether acetate) was applied for 20 min to dissolve and remove the unexposed photoresist. Finally, the sample was rinsed in isopropyl alcohol for 4 min and dried with nitrogen. More details are provided in the Supplementary Materials (Section S8).

### 6.3 Measurements

**Figure 8:**
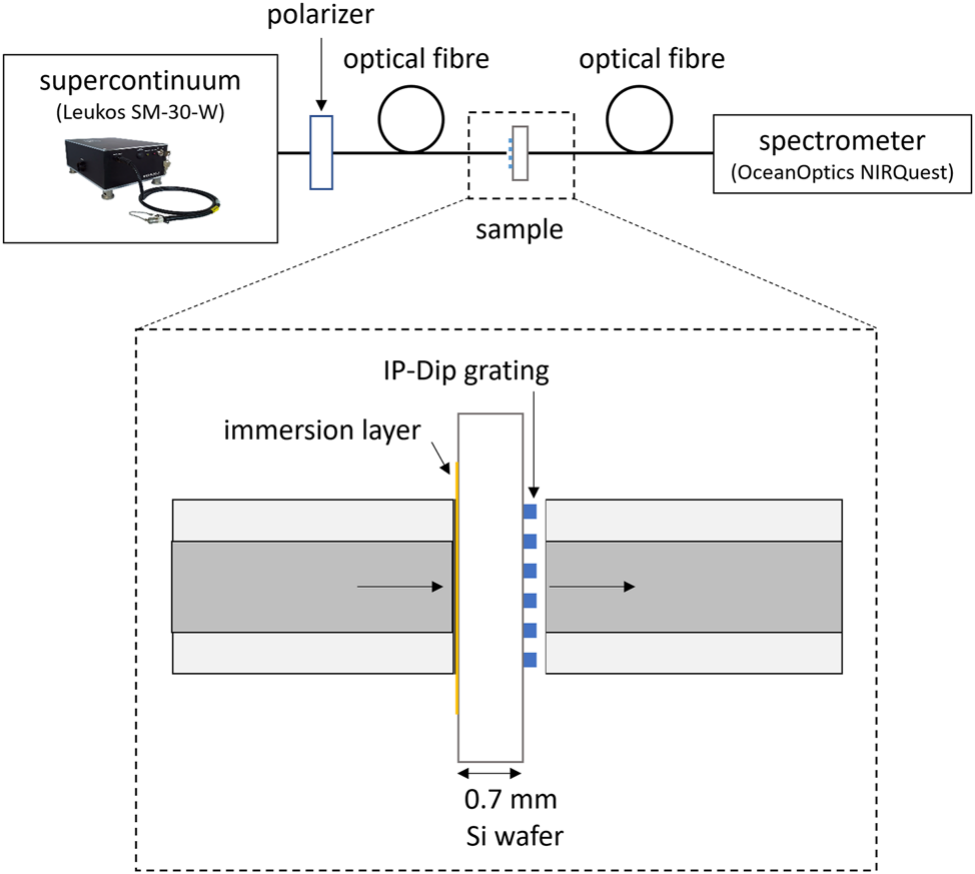
Diagram of the experimental setup.

For the transmission measurements, a supercontinuum light source (Leukos SM-30-W; 400–2400 nm) was coupled to the optical fiber, illuminating the sample from the side. The polarizer for 1550 nm was placed between the supercontinuum source and the sample and a rotary stage was used to change the angle of polarization. On the opposite side of the sample, a detection optical fiber was moved precisely toward the sample using an immersion layer. The transmission spectra of the ICG grating were measured using an OceanOptics NIRQuest spectrometer (900–2050 nm) with respect to the reference transmission of the silicon substrate. Figure 8 shows a diagram of the experimental setup.

## Supplementary Material

Supplementary Material Details
