# Supplementary material for: Concept of inverted refractive-index-contrast grating mirror and exemplary fabrication by 3D laser micro-printing
Source: Nanophotonics. 2023 Aug 29;12(18):3579–88. doi: 10.1515/nanoph-2023-0283 (PMC11501227; doi:10.1515/nanoph-2023-0283)
Supplement: Supplementary file 1 — Supplementary Material Details [file j_nanoph-2023-0283_suppl_001.pdf]

Supplementary Information to "Concept of Inverted Refractive-Index-Contrast Grating Mirror and Exemplary Fabrication by 3D Laser Micro-Printing"

Emilia Pruszyńska-Karbownik,[1] Daniel Jandura,[2] Maciej Dems,[3] Łukasz Zinkiewicz,[1] Artur Broda,[4] Marcin Gębski,[5] Jan Muszalski,[4] Dušan Pudiš,[2,6] Jan Suffczyński,[1,*] and Tomasz Czyszanowski[3,†]

[1]*Institute of Experimental Physics, Faculty of Physics, University of Warsaw, ul. Pasteura 5, 02-093 Warsaw, Poland*
[2]*Department of Physics, Faculty of Electrical Engineering and Information Technology, University of Žilina, Univerzitna 1, SK-01026 Žilina, Slovakia*
[3]*Institute of Physics, Lodz University of Technology, ul. Wólczańska 217/221, 93-005 Łódź, Poland*
[4]*Łukasiewicz Research Network, Institute of Microelectronics and Photonics, al. Lotników 32/46, 02-668 Warsaw, Poland*
[5]*Institute of Physics, Łódz University of Technology, ul. Wólczańska 217/221, 93-005 Łódź, Poland*
[6]*University Science Park of the University of Žilina, Univerzitna 1, SK-01026 Žilina, Slovakia;*

This file contains supplementary information to the article "Concept of Inverted Refractive-Index-Contrast Grating Mirror and Exemplary Fabrication by 3D Laser Micro-Printing".

S1. DESCRIPTION OF THE PROPERTIES OF TYPE 2 REFLECTIVITY

The peculiar behavior of the Type-2 reflection peak — its independence of the refractive index of the cladding — is a consequence of the relation between the electric and magnetic field at the reflection side of the grating [S1]. Let us consider a zero-order TE-polarized plane wave incident from the substrate side, traveling into a perfect infinite grating. It is sufficient to consider only individual components of the electric and magnetic fields parallel to the grating (E_y and H_x). The normalized fields in the cladding at the grating boundary are

$$E^{(1)}(x,z) = 1 + \sum_n r_n \cos(2\pi n x/L), \tag{S1a}$$

$$H^{(1)}(x,z) = \alpha_0 - \sum_n r_n \alpha_n \cos(2\pi n x/L), \tag{S1b}$$

where n is the number of the diffraction order, r_n is the amplitude reflection coefficient of the n-th diffraction order, and α_n is

$$\alpha_n = \sqrt{\varepsilon_c - (n\lambda/L)^2}, \tag{S2}$$

where $\varepsilon_c = n_c^2$ is the cladding permittivity.

To eradicate x-dependence from the above equations, the equations are multiplied by $\cos(2\pi m x/L)$ and integrated over the grating period. This allows vectors of consecutive orders of the electric ($\mathbf{e}$) and magnetic ($\mathbf{h}$) fields to be represented as

$$\mathbf{e} = \mathbf{d}_0 + \mathbf{r}, \tag{S3a}$$

$$\mathbf{h} = \boldsymbol{\alpha}\left(\mathbf{d}_0 - \mathbf{r}\right), \tag{S3b}$$

where $\mathbf{d}_0 = \begin{bmatrix} 1 & 0 & 0 & \dots \end{bmatrix}^T$ is Kronecker's delta vector, $\mathbf{r}$ is the vector of the reflection coefficients, and $\boldsymbol{\alpha}$ is the diagonal matrix with elements defined by Eq. (S2). It is possible to derive a relation between $\mathbf{e}$ and $\mathbf{h}$ using only grating parameters, independently of the cladding refractive index in the form [S1]

$$\mathbf{h} = \mathbf{Z}\,\mathbf{e}. \tag{S4}$$

The matrix $\mathbf{Z}$ is the input impedance matrix. This yields the reflection of the system as

$$\mathbf{r} = \left(\boldsymbol{\alpha} + \mathbf{Z}\right)^{-1}\left(\boldsymbol{\alpha} - \mathbf{Z}\right)\mathbf{d}_0. \tag{S5}$$

---

* j.suffczynski@uw.edu.pl
† tomasz.czyszanowski@p.lodz.pl

For the Type-2 high reflectivity peak, the diagonal elements of the impedance matrix dominate over non-diagonal elements. Furthermore, the zero-order element Z_{00} is purely imaginary. In such case, the zero-order reflection coefficient is (from Eqs. (S2) and (S5))

$$r_0 \approx \frac{\varepsilon_c - Z_{00}}{\varepsilon_c + Z_{00}}. \tag{S6}$$

As Z_{00} is purely imaginary, $|r_0| \cong 1$ regardless of the value of ε_c. In other words, the zero-order reflectivity coefficient is always 100% regardless of the value of n_c, which only impacts the phase of the reflected wave. In accordance with the law of conservation of energy, all other reflected diffraction orders are suppressed.

For small n_g (less than 1.75) the $|r_0|$ and $|r_1|$ diagonal elements are no more negligible. This indicates the existence of zeroth and first diffraction orders in the reflection, precluding 100% reflection into the zeroth diffraction order.

S2. NUMERICAL DETERMINATION OF MODES DISPERSION BY THE LIGHT CONFINEMENT IN THE GRATING

In this section and the section that follows we consider dependence of the resonant wavelength of the modes on the height of the grating stripes and this dependence for brevity is called dispersion. Numerical identification of the dispersion curves of the grating modes using an eigenvalue solver based on the plane wave admittance method (PWAM) [S2] is significantly hindered by the very low Q-factor of the leaky modes in an ICG, which become untraceable for the solver. Therefore, we identify the modes using the method described in [S3], whereby the modes are identified by the build-up of light intensity inside the ICG as minima according to the formula

$$D = \frac{\partial^2}{\partial H^2}\Im = \frac{\partial^2}{\partial H^2}\frac{H_c \int_{\mathrm{ICG}} EE^*\, dy\, dz}{H \int_{\mathrm{c}} EE^*\, dy\, dz} \tag{S7}$$

where E is the electric component of the electromagnetic field that is determined by the plane-wave reflection transformation method (PWRTM) described in the "Numerical methods" section in the main text. The integral in the nominator is over the ICG layer with thickness H and the integral in the denominator is over the cladding layer with H_c, which is significantly larger than the wavelength in the cladding. By $\Im$ is designated the relative light confinement in the ICG. Figure S1 compares the dispersion curves of TE_{20}, TE_{21}, and TE_{22} modes in a grating membrane composed of stripes with a refractive index of 2 surrounded by air. In such a membrane configuration, the quality (Q) factor of the modes is high enough to be traceable by PWAM and therefore enables direct comparison with the method based on $\Im$ determination by PWRTM. The dispersion curves determined by PWRTM in Fig. S1a and by PWAM in Fig. S1b have no perceptible differences. The points composing the curves display colours corresponding to $\Im$ in Fig. S1a and to Q-factor in Fig. S1b. Local maxima of the Q-factor relate to interference-based bound states in the continuum (BIC) occurring in vertically symmetric gratings [S4]. Symmetry-protected BICs are absent in the figures, due to the boundary conditions in PWAM enabling laterally symmetric modes only that emit zeroth diffraction order under normal direction. Therefore normal incidence considered in the PWRTM enables coupling the light to the symmetric grating modes only. The positions of the local maxima of Q and $\Im$ are also located for the same grating parameters, which results from the fact that an increase in the Q-factor accompanies an increase in the density of the optical field inside the cavity.

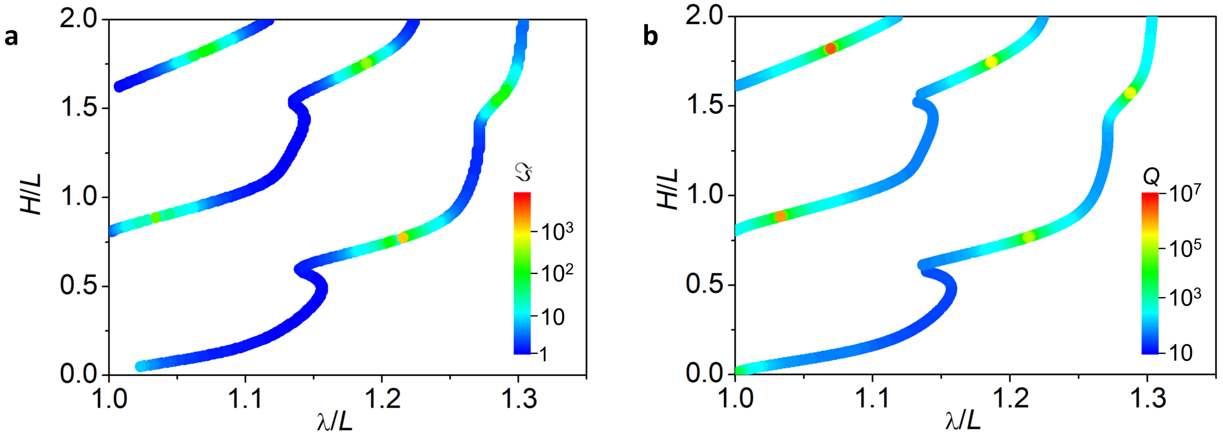

Figure S1. Mode dispersion in the domain of wavelength λ and grating height H both relative to the grating period L calculated by PWRTM in a) and PWAM in b). The refractive index of the grating n_g is 2 and refractive index of the surroundings is 1. The fill factor F of the grating stripes is 0.4. Colors represent relative light confinement $\Im$ in ICG in a) and Q-factor in b).

S3. NUMERICAL DETERMINATION OF LEAKY MODES DISPERSION BY THE LIGHT CONFINEMENT IN THE ICG

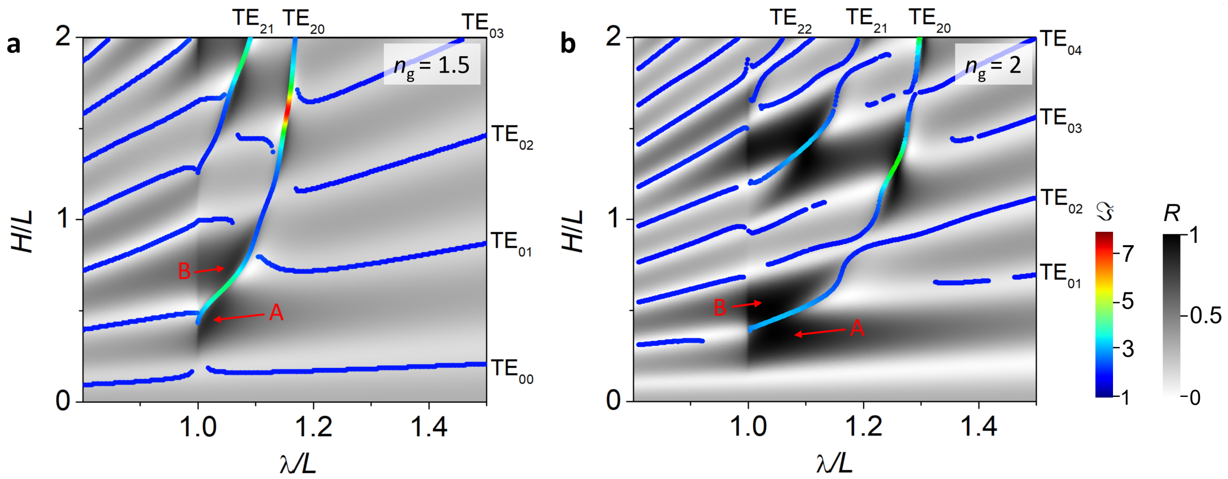

Figure S2. Reflectance (R) maps displayed in grey scale for the inverted contrast grating (ICG) in the domain of wavelength λ and grating height H both relative to the grating period L. The refractive index of the cladding n_c is 3.5. The refractive index of the grating and fill factor are $n_g = 1.5$, $F = 0.397$ in a) and $n_g = 2.0$, $F = 0.447$ in b). The colour lines in a) and b) represent the dispersion of ICG modes and their relative light confinement in the ICG ($\Im$) as defined in Section S2. Positions of reflection maxima A and B are indicated by arrows.

Figures S2 demonstrate the reflectivity maps of ICG configurations that are also presented in Fig. 4 in main text with overlapped dispersion of leaky modes existing in the structures calculated by the method described in section S2. PRMs A and B are positioned in the proximity of the dispersion of TE_{20} mode that affects the light distribution in the case of both maxima. We also determine the relative light confinement $\Im$ of the modes that is indicated by color. In the case of TE_{20} mode there is a significant build-up of light density in the grating reaching an 8-fold increase for $n_g = 1.5$ and a 5-fold increase for $n_g = 2.0$ compared to the light density in the cladding.

S4. PHASE OF REFLECTED LIGHT AND POLARIZATION DISCRIMINATION

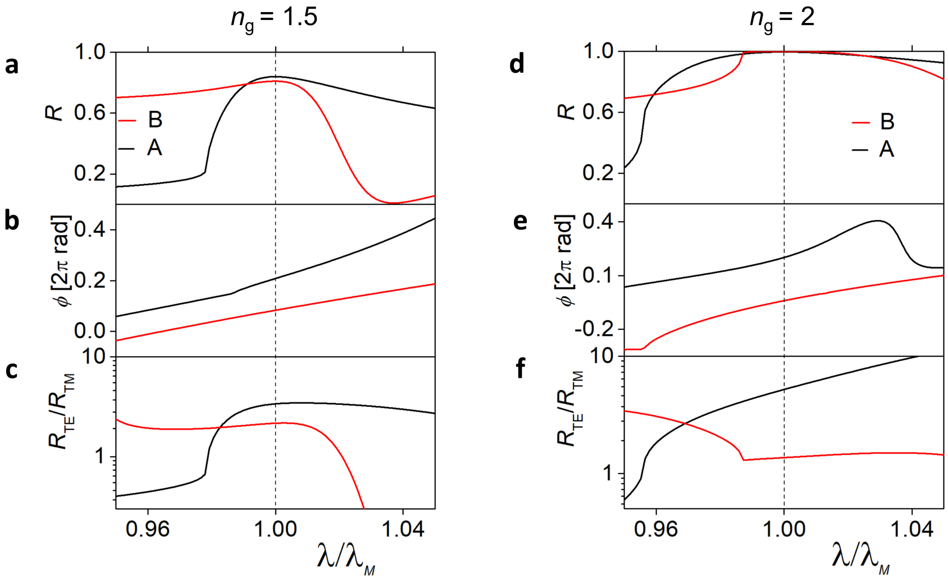

Figure S3. Spectral dependences of a), d) reflection, b), e) phase of the reflected light, c), f) ratio of optical power reflectance between TE and TM polarizations, for inverted refractive index contrast grating (ICG) configurations corresponding to B and A PRMs indicated by red and black, respectively. Details of the configurations are summarized in Table S1. Wavelength is unitless and $\lambda = 1$ represents the central wavelength corresponding to maximal optical power reflectance.

Figure S3 summarizes the principal properties characterizing the performance of the considered gratings for normal incidence of light. The spectrum shown in Fig. S3d for an ICG with $n_g = 2$ indicates maximal power reflectance of nearly 100%. The reflectance decreases to 85% when n_g decreases to 1.5, as illustrated in Fig. S3a. Figures S3b and e show that the considered gratings ensure efficient tuning of the phase (ϕ) of the reflected light. In both cases, corresponding to $n_g = 1.5$ and $n_g = 2$, $d\phi/d\lambda \approx 10\pi$ rad at the vicinity of the reflection maximum. This property provides a facile method of tuning the resonant wavelength of a cavity with an ICG mirror, by modifying the geometrical parameters of the ICG while keeping the cavity thickness constant [S5]. Figures S3c and f demonstrate the ratio of reflectance of TE to TM polarized light. The level of polarisation discrimination is similar to that achieved previously with HCGs and MHCGs [S6–S9]. Several times larger power reflectance for TE polarization with respect to TM could allow strong polarization discrimination of the stimulated emission when an ICG is applied as a mirror in a Fabry-Perot cavity.

S5. DISPERSION OF THE LIGHT IN REFLECTION AND TRANSMISSION

To characterize the dispersion of transmission and reflection of the light incident on the grating from the bottom side, we calculate both values as a function of λ/L and k_x/k_0, where k_x is the in-plane x component (see Fig. 1 in the main text) of the incident photon momentum and k_0 is the wavenumber of the light in a vacuum. Calculated transmission and reflection are shown in the left and right panels of the subplots in Fig. S4. The maps illustrate a selected range of the plane (k_x/k_0, λ/L), in the vicinity of $k_x = 0$ corresponding to normal incidence. The junction of the second and third photonic bands (PB) lies in the center of the first Brillouin zone [S10]. The reflection is calculated for the zeroth diffraction order for the incidence from the cladding side, whereas the transmission is calculated for all diffraction orders and for light incident from the air side. This choice is motivated by the fact that in the case of a vertical cavity with an ICG as a mirror, only the zeroth diffraction order of the reflected light is involved in Fabry-Perot resonance, whereas in the case of external optical excitation the total transmission of light through the mirror may contribute to excitation of the active material embedded in the cavity. As can be seen in Fig. S4, high reflectivity closely follows the folded branch of the light line in the subwavelength regime. Comparison of the reflection panels in Figs. S4a and S4b indicate that the high reflectivity region widens with respect to k_x when n_g increases from 1.5 to 2. The presence of an abrupt transition between high and low reflectivity regions for $n_g = 1.5$ suggests that the high reflection is due to Fano-like resonance. A build-up of light intensity is also observed in Fig. 4c in the main text, which supports the hypothesis that Fano resonance is responsible for the strong reflection in the case of gratings with a lower refractive index. Analysis of the transmission panels indicates that the transmission of the ICG is relatively high and uniform in the analyzed range of k_0 and k_x values. It is additionally enhanced in proximity to the folded branches of the light line.

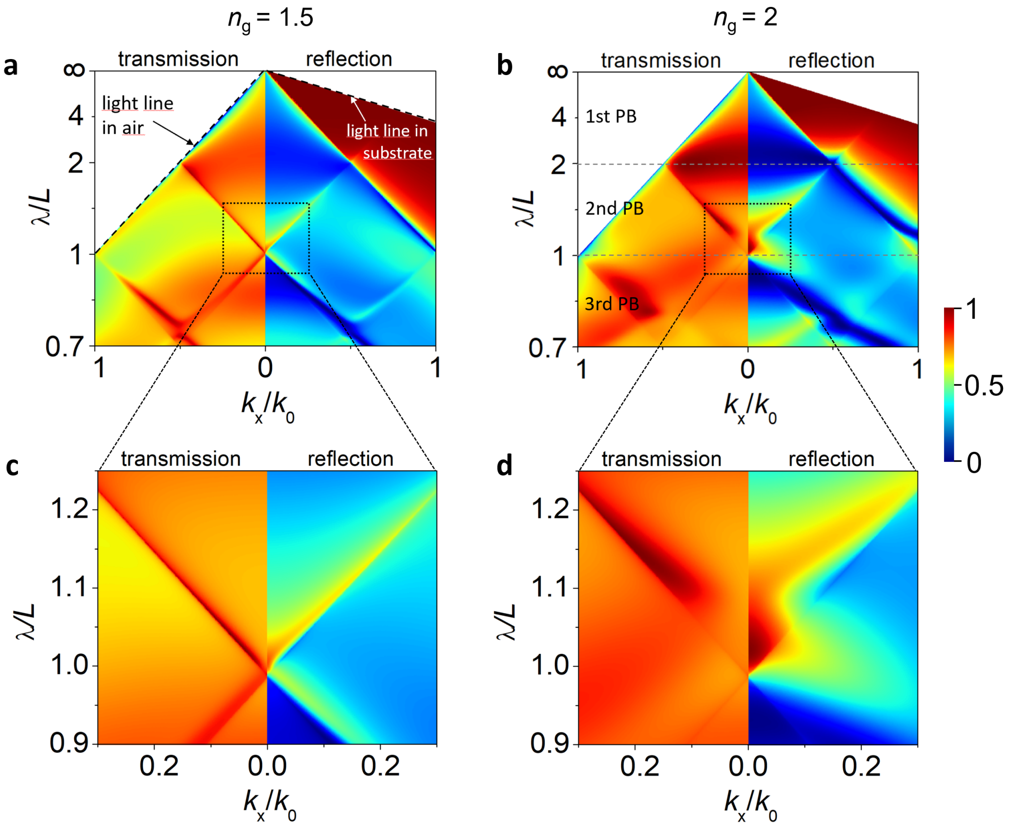

Figure S4. Dispersion diagrams of transmission (left panels) and reflection (right panels) for configurations of inverted refractive index contrast gratings defined by a grating refractive index $n_g = 1.5$, fill factor $F = 0.395$, stripe height $H/L = 0.468$ in a), c) corresponding to configuration (1) in Fig. 4a in the main text and a grating refractive index of $n_g = 2.0$, fill factor $F = 0.414$, stripe height $H/L = 0.373$ in b), d) corresponding to configuration (3) in Fig. 4b in the main text. In a) the borders between the photonic bands (PB) are indicated with horizontal dashed lines. In b) the light lines in the cladding and air are indicated with dashed lines. The rectangular black dotted lines in a) and b) indicate the borders of the regions illustrated in c) and d)
.

S6. REFLECTION INTO ALL DIFFRACTION ORDERS AND POWER REFLECTANCE SPECTRA FOR ARBITRARY n_g

Figure S5 illustrates ICG reflection for variable n_g and $n_c = 3.5$ in the cases of two local reflection maxima (A, B) for the smallest H. The local maxima are found using multidimensional optimization, including L, F, and H of the ICG as variables. Slowly varying the positions of A and B maxima with respect to the grating parameters when n_g is modified ensures that both maxima can be tracked carefully. The geometrical parameters of the maxima A and B corresponding to various n_g are collected in Tab. S1. Maximum B (Fig. S5b) exhibits above 0.99 power reflectance into the zeroth diffraction order in the range of n_g from around 1.75 to 3.0 and total reflection above $1 - 10^{-4}$ in the range of n_g from 1.8 to 2.8. For $n_g = 1.9$ reflection into the zeroth diffraction order reaches the value of 1 with an accuracy of 10^{-8}. High reflection into the zeroth diffraction order is possible due to the reduction of light reflection into higher diffraction orders, as described in Section II of the main text and Section S1 in the Supplementary Materials. Maximum A (Fig. S5a) reveals a broader n_g range of above 0.99 reflectance into the zeroth diffraction order in comparison to maximum B, ranging from around 1.75 to 3.5. This is the limit for an ICG when assuming $n_c = 3.5$. In a very similar range from 1.8 to 3.5, total power reflectance is above $1 - 10^{-4}$, with the exception of the range from 2.8 to 3.25 where it reduces to 0.996.

Figures S5c and d are composed of zeroth diffraction order reflectance spectra of maxima B and A as for different grating refractive index (n_g) modifying from 1.1 to 3.5 with 0.3 step. Figure S5c indicates the tendency of the width of reflection stopband (WRS, which we define as above 60% of the reflection stopband) to narrow as n_g reduces. The WRS of the B maximum is significantly narrower with "sharp" feature in the proximity of the maximum. Therefore, the B maximum can be considered as a possible narrowband filter while the A maximum shows features that may be useful for mirrors in Fabry-Perot resonators.

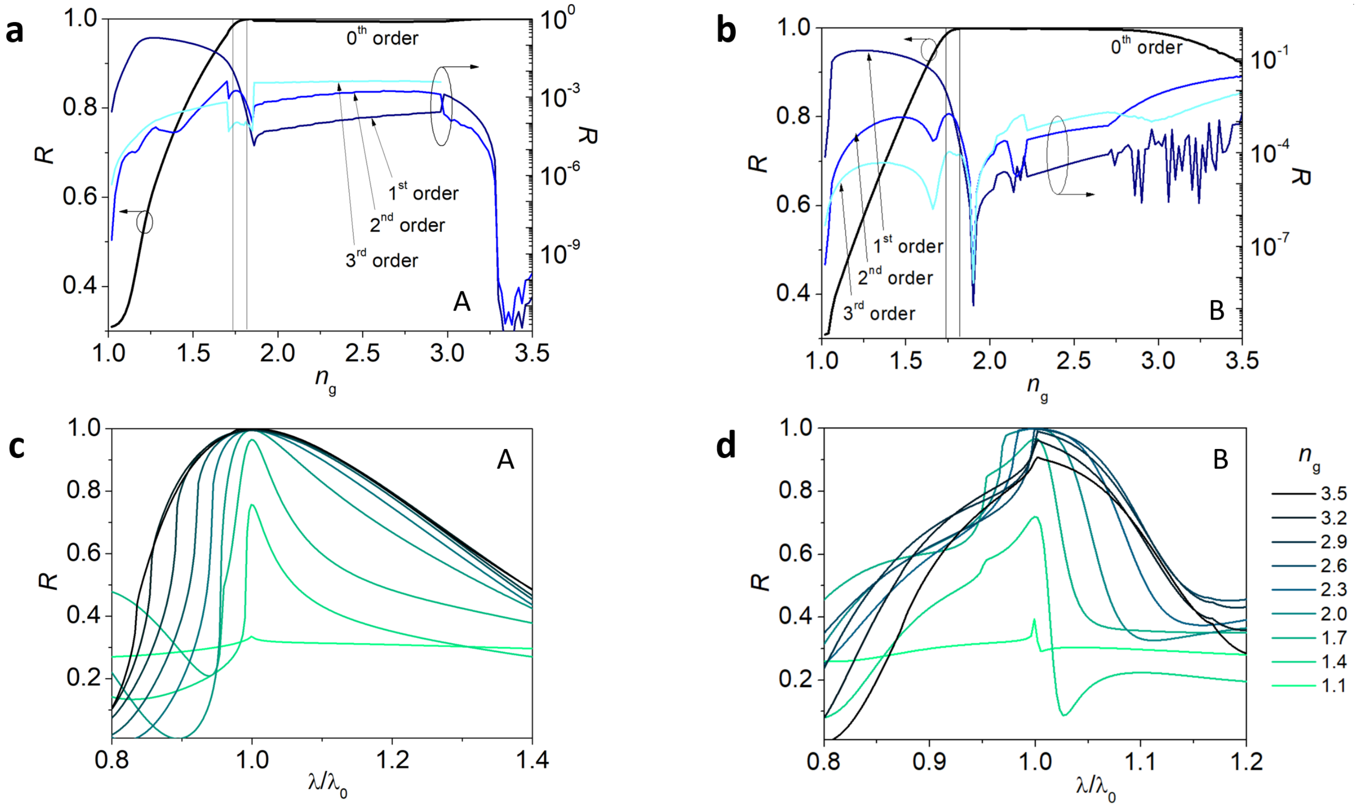

Figure S5. ICG power reflectance into all diffraction orders as functions of the refractive index of the ICG grating n_g in the case of A a) and B b). Geometrical parameters of ICGs corresponding to reflection maxima are collected in Tab. S1. In c) and d), reflectance spectra are shown corresponding to maxima A and B, respectively, for n_g modified in the range from 1.1 to 3.5 with 0.3 step indicated by colors.

Table S1. Geometrical parameters of the ICG for configurations corresponding to A and B maxima for various refractive index of the grating n_g: L – period of the grating, F – fill factor, H – height of the stripe and for $n_c = 3.5$.

		A			B	
n_g	λ/L	F	H/L	λ/L	F	H/L
1.1	1.000	0.452	0.532	0.998	0.471	0.952
1.2	1.000	0.430	0.516	0.980	0.450	0.905
1.3	0.999	0.421	0.461	0.962	0.430	0.846
1.4	0.991	0.408	0.458	0.951	0.413	0.782
1.5	0.978	0.395	0.458	0.946	0.397	0.714
1.6	0.967	0.388	0.463	0.947	0.383	0.644
1.7	0.957	0.382	0.485	0.952	0.374	0.563
1.8	0.958	0.397	0.519	0.958	0.397	0.519
1.9	0.958	0.423	0.399	0.964	0.431	0.503
2	0.956	0.414	0.357	0.969	0.447	0.486
2.1	0.952	0.406	0.326	1.000	0.370	0.496
2.2	0.948	0.398	0.302	1.000	0.415	0.462
2.3	0.942	0.391	0.283	0.981	0.462	0.430
2.4	0.936	0.384	0.266	0.987	0.460	0.414
2.5	0.929	0.378	0.252	0.993	0.455	0.398
2.6	0.921	0.373	0.239	0.998	0.449	0.383
2.7	0.912	0.367	0.228	1.000	0.443	0.369
2.8	0.903	0.363	0.218	1.000	0.437	0.356
2.9	0.892	0.359	0.209	1.000	0.429	0.346
3	0.857	0.376	0.201	1.000	0.421	0.335
3.1	0.857	0.369	0.193	1.000	0.412	0.327
3.2	0.857	0.363	0.186	1.000	0.404	0.319
3.3	0.857	0.356	0.180	1.000	0.396	0.313
3.4	0.847	0.354	0.174	1.000	0.388	0.303
3.5	0.836	0.351	0.168	1.000	0.381	0.300

S7. CALCULATION OF THE OPTICAL PROPERTIES OF A REAL-WORLD IHCG 3D MICRO-PRINTED USING IP-DIP

Figures S6a and S6b show SEM images of the fabricated ICG. The cross-sectional shapes of the stripes exhibit a non-rectangular cross section, which we attribute to interference of the laser light in the vicinity of the substrate surface and to the high susceptibility of the two-photon absorption process to the laser power. In both images, the original scale is presented to enable determination of the lateral dimensions of the ICG. In Fig. S6c, results of profilometer measurements are shownm, indicating the very high repeatability of the stripe height.

Figure S7a shows a reflectance map of the ICG calculated for the experimental cross-sectional shape of the grating shown in Fig. 6c of the main text and illuminated by TE polarisation at normal incidence from the Si substrate (bottom) side. The nonrectangular cross section of the ICG stripes is responsible for the more than 100 nm shift in the optimal H toward smaller values compared to the rectangular cross section. Figure S7b illustrates the intensity of light incident from the substrate side, indicating a significant build-up of light intensity in the grating. The pattern resembles the distribution of light in the rectangular cross-section illustrated in Fig. 4c.

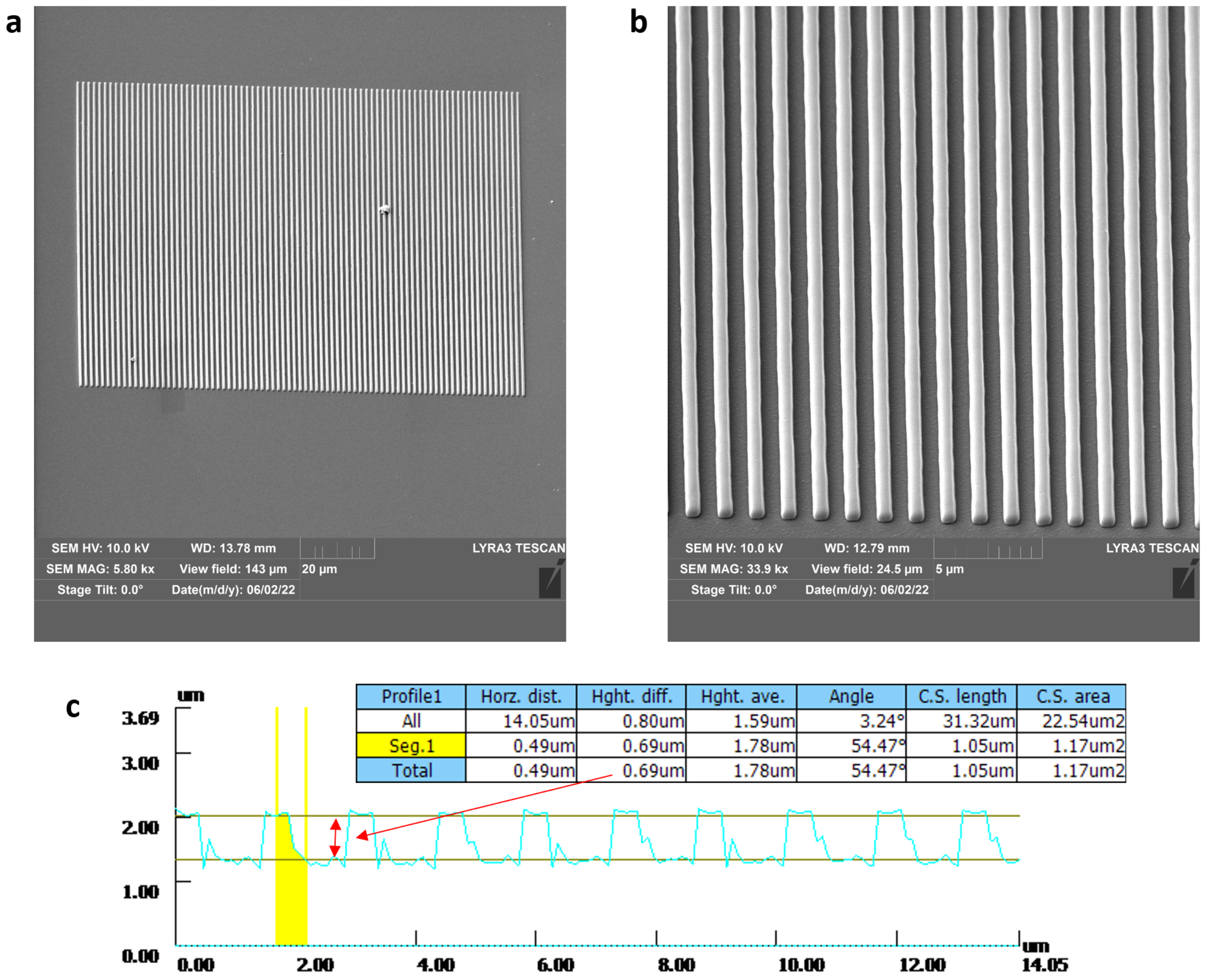

Profile1	Horz. dist.	Hght. diff.	Hght. ave.	Angle	C.S. length	C.S. area
All	14.05um	0.80um	1.59um	3.24°	31.32um	22.54um2
Seg.1	0.49um	0.69um	1.78um	54.47°	1.05um	1.17um2
Total	0.49um	0.69um	1.78um	54.47°	1.05um	1.17um2

Figure S6. SEM images of IP-Dip inverted refractive index contrast gratings (ICG): a), b) top-down view images of ICG stripes with different magnifications, images taken at an angle of 45 degrees to the plane of the wafer; c) profile of the ICG measured using a confocal microscope.

S8. ADDITIONAL DETAILS REGARDING FABRICATION PROCESS OF SUBWAVELENGTH GRATINGS BY 3D MICRO-PRINTING

In principle, there is no limitation for the volume of processed structures (max. area is approx. 100×100 mm^2). However, the structures with dimensions over 200 μm $\times$ 200 μm $\times$ 200 μm need to be prepared by a combination of linear and piezo movement controllers. So, the stitching of individual parts is necessary and the stitching creates some discontinuities (see, e.g., [S11]). The optimal area of the grating is up to 200 μm $\times$ 200 μm. With the scanspeed of 10000 μm/s the time for our grating fabrication (120 μm $\times$ 120 μm) was 38 s. The precision of the writing process is tens of nanometers, so the uniformity oscillates in this range.

The degree of polymerization of IP-Dip and hence its refractive index, depend on a number of parameters such as total UV dose or heat treatment subsequent to the polymerization (see, e.g., [S12]). According to the literature, the refractive index of two-photon polymerized IP-Dip ranges from 1.525 to 1.535 for the wavelength of 1550 nm related to processing and post-processing. In our case, we assumed that the refractive index of polymerized IP-Dip is equal 1.530.

In the case of objects micro-printed using the IP-Dip a so-called downscaling can be also utilized. It relies on annealing of an object to induce lowering of its size and modification of its chemical composition, thus also its

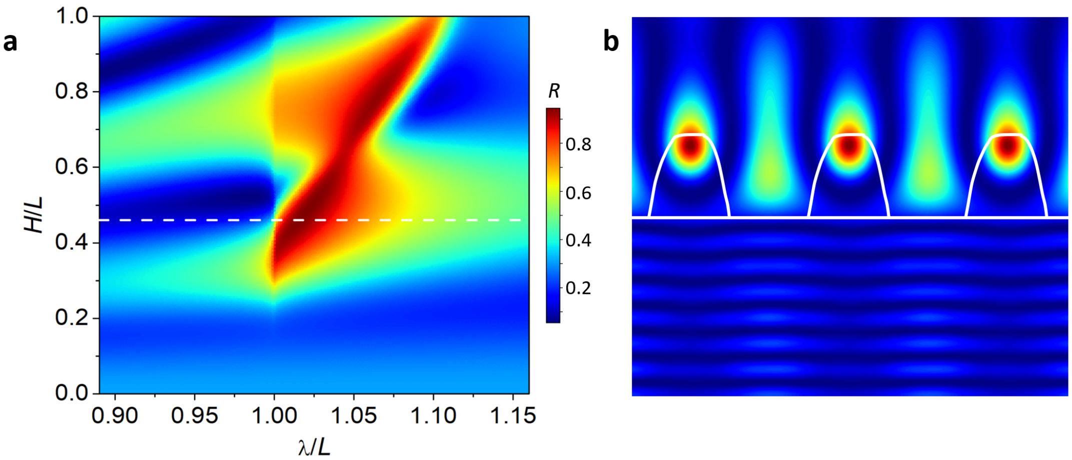

Figure S7. a) Calculated reflectance map for IP-Dip inverted refractive index contrast gratings (ICGs) for the cross section of the stripes illustrated in b), for $F = 0.45$ in the domain of the wavelength λ, and for the grating height H. The horizontal white dashed line represents $H/L = 0.46$. In b) is shown the distribution of optical field intensity within the IP-Dip ICG of the real-world cross section when illuminated by a plane wave at normal incidence from the cladding side.

refractive index (see, e.g., [S13]). In our case the downscaling is not necessary as the grating parameters ($L = 1460$ nm, $a = 670$ nm) are achievable by a standard writing process. We note here that the IP-Dip experiences a well-known shrinkage effect after polymerization (around 5-17%). In our case, the gratings were deposited on the planar basis on Silicon cladding, so the shrinkage was extremely small and the parameters of the structures remained as intended.

[S1] Dems, M. Monolithic high-contrast gratings: why do they not scatter light? *Journal of Lightwave Technology* **35**, 159–165 (2017).

[S2] Dems, M., Kotynski, R. & Panajotov, K. Planewave admittance method — a novel approach for determining the electromagnetic modes in photonic structures. *Opt. Express* **13**, 3196–3207 (2005).

[S3] Marciniak, M. *et al.* Impact of stripe shape on the reflectivity of monolithic high contrast gratings. *ACS Photonics* **8**, 3173–3184 (2021).

[S4] Hsu, C. W., Zhen, B., Stone, A. D., Joannopoulos, J. D. & Soljačić, M. Bound states in the continuum. *Nature Reviews Materials* **1**, 16048 (2016).

[S5] Haglund, E. *et al.* Demonstration of post-growth wavelength setting of VCSELs using high-contrast gratings. *Optics Express* **24**, 1999 (2016).

[S6] Chang-Hasnain, C. J. & Yang, W. High-contrast gratings for integrated optoelectronics. *Adv. Opt. Photonics* **4**, 379–440 (2012).

[S7] Gębski, M., Lott, J. A. & Czyszanowski, T. Electrically injected VCSEL with a composite DBR and MHCG reflector. *Optics Express* **27**, 7139 (2019).

[S8] Marciniak, M. *et al.* Tuning of reflection spectrum of a monolithic high-contrast grating by variation of its spatial dimensions. *Optics Express* **28**, 20967–20977 (2020).

[S9] Hong, K.-B. *et al.* Monolithic high-index contrast grating mirror for a GaN-based vertical-cavity surface-emitting laser. *Photonics Research* **9**, 2214 (2021).

[S10] Joannopoulos, J. D., Johnson, S. G., Winn, J. N. & Meade, R. D. *Photonic Crystals: Molding the Flow of Light - Second Edition* (Princeton University Press, 2008), rev - revised, 2 edn.

[S11] Stein, O. *et al.* Fabrication of low-density shock-propagation targets using two-photon polymerization. *Fusion Science and Technology* **73**, 153–165 (2018).

[S12] Schmid, M., Ludescher, D. & Giessen, H. Optical properties of photoresists for femtosecond 3d printing: refractive index, extinction, luminescence-dose dependence, aging, heat treatment and comparison between 1-photon and 2-photon exposure. *Opt. Mater. Express* **9**, 4564–4577 (2019).

[S13] Sharipova, M. I. *et al.* Effect of pyrolysis on microstructures made of various photoresists by two-photon polymerization: comparative study. *Opt. Mater. Express* **11**, 371–384 (2021).
